# Left-sided Inferior Vena Cava with Hemiazygos Continuation to Left Superior Vena Cava

**DOI:** 10.7759/cureus.6503

**Published:** 2019-12-29

**Authors:** Ismail Kabakus, Madison Kocher, Ali Agha, Jeremy R Burt

**Affiliations:** 1 Radiology, Medical University of South Carolina, Charleston, USA; 2 Internal Medicine, University of Texas Health Science Center, Houston, USA; 3 Radiology/Cardiothoracic, Medical University of South Carolina, Charleston, USA

**Keywords:** persistent left superior vena cava, left inferior vena cave, congenital anomaly

## Abstract

Congenital anomalies of superior (SVC) and inferior vena cava (IVC) are not uncommon and usually incidentally recognized. The normal embryogenesis is a complex process involving the formation of several anastomoses. Failure of certain vessels to develop or regress results in numerous caval variations and anomalies. Although these are usually without significant clinical implications, awareness of these anomalies is necessary to avoid diagnostic pitfalls and suggest the presence of other abnormalities and for the planning of vascular intervention or surgery. We present a very rare, caval anomaly, a left-sided IVC with hemiazygos continuation to left SVC in the absence of right SVC.

## Introduction

Congenital variations or anomalies of venae cavae are not uncommon with a reported prevalence up to 8.7% [1]. These anomalies are usually incidentally recognized. They may be associated with other anomalies, especially cardiac. The normal embryogenesis is a complex process involving the formation of several anastomoses. Failure of certain vessels to develop or regress results in numerous caval variations and anomalies [2-3]. Although these are usually without significant clinical implications, awareness of these anomalies is necessary to avoid diagnostic pitfalls and is important pre-operatively.

Common superior vena cava (SVC) anomalies are left-sided and duplicated SVC. Inferior vena cava (IVC) anomalies can be classified as pre-renal (interrupted IVC), renal (retroaortic renal vein and circumaortic venous callor) and post-renal (duplicated IVC, left-sided IVC and retro caval ureter) [1].

In this case report, we briefly review the embryogenesis of SVC and IVC and present a very rare case with both SVC and IVC anomalies. 

## Case presentation

**Figure 1:**
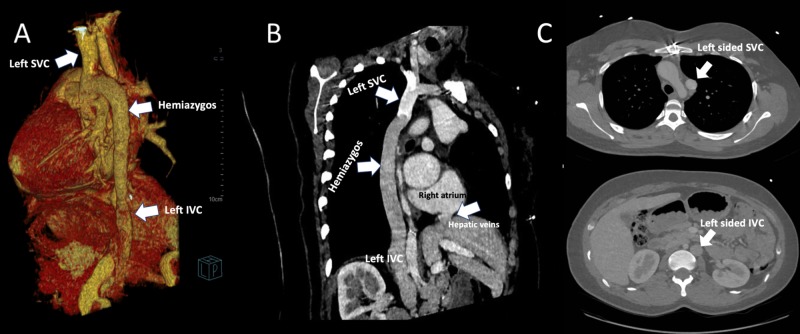
Volume rendered and maximum intensity CTA A) Volume rendered chest CTA showing the left IVC with hemiazygos continuation draining to the left-sided SVC. (B) Oblique coronal maximum intensity CTA image of the chest demonstrating the left hemiazygous arch. The hepatic veins drain directly into the right atrium. The branch from the left SVC draining into the coronary sinus is not included in the image. (C) Axial CTA images showing left-sided SVC and left-sided IVC. CTA, computed tomography angiography; SVC, superior vena cava; IVC, inferior vena cava

A 30-year-old asymptomatic female with a history of surgically repaired aortic coarctation presented for routine follow-up. Chest computed tomography angiography (CTA) demonstrated a left-sided IVC with hemiazygos continuation to the left-sided SVC in the absence of right-sided SVC (Figure 1A), a very rare systemic venous anomaly. The left-sided superior vena cava was draining to the enlarged coronary sinus, and the hepatic veins were directly connected to the right atrium (Figure 1B). 

The case has been followed up for the coarctation without any treatment regarding to venous anomaly.

## Discussion

**Figure 2:**
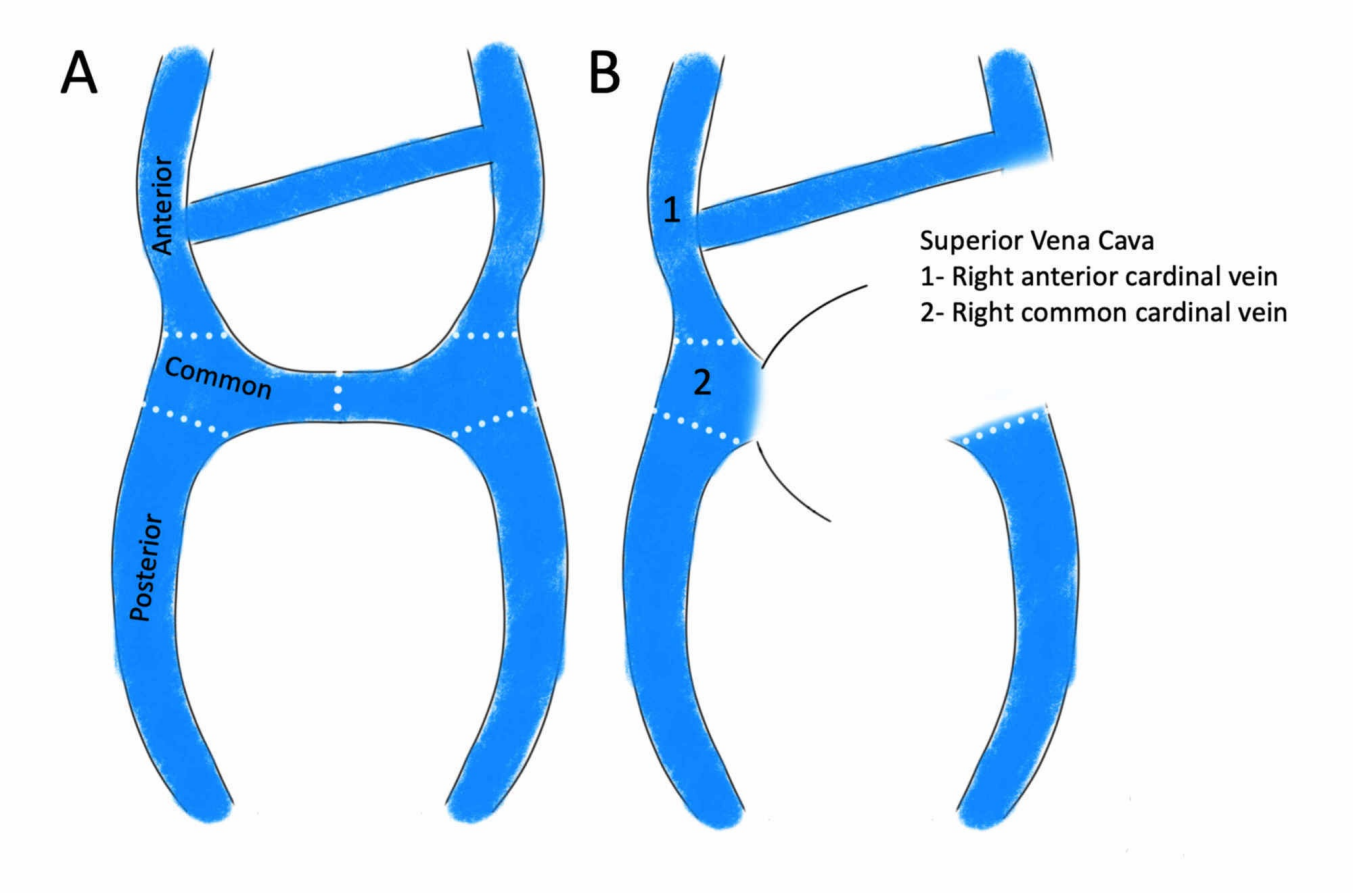
Normal embryological formation of SVC (A) Pictorial depiction of the oblique vein anastomosis between the two anterior cardinal veins. (B) Normal regression of the left-sided major veins. With this regression, the anastomotic vein between the two becomes left brachiocephalic (innominate) vein and right anterior and right common cardinal veins form the SVC. SVC, superior vena cava

The embryogenesis of SVC starts with two major symmetrical vein systems, right and the left anterior cardinal veins draining the upper portion of the embryo. Each anterior cardinal vein drains into a common cardinal vein connected to the embryological heart, and an oblique vein anastomosis forms between the two anterior cardinal veins (Figure 2A). Due to the physiological right-to-left shunt during embryological life, right-sided veins develop and left-sided major veins regress. The left common cardinal vein regresses and becomes the ligament of Marshall, and the anastomotic vein between the two anterior cardinal veins becomes the left brachiocephalic (innominate) vein, while the right anterior and right common cardinal veins form the SVC (Figure 2B) [2]. Failure of left common cardinal vein regression results in a persistent left SVC, the most common congenital anomaly involving the SVC [2]. If the right common cardinal vein regresses, there is only a left-sided SVC, as seen in our case.

**Figure 3:**
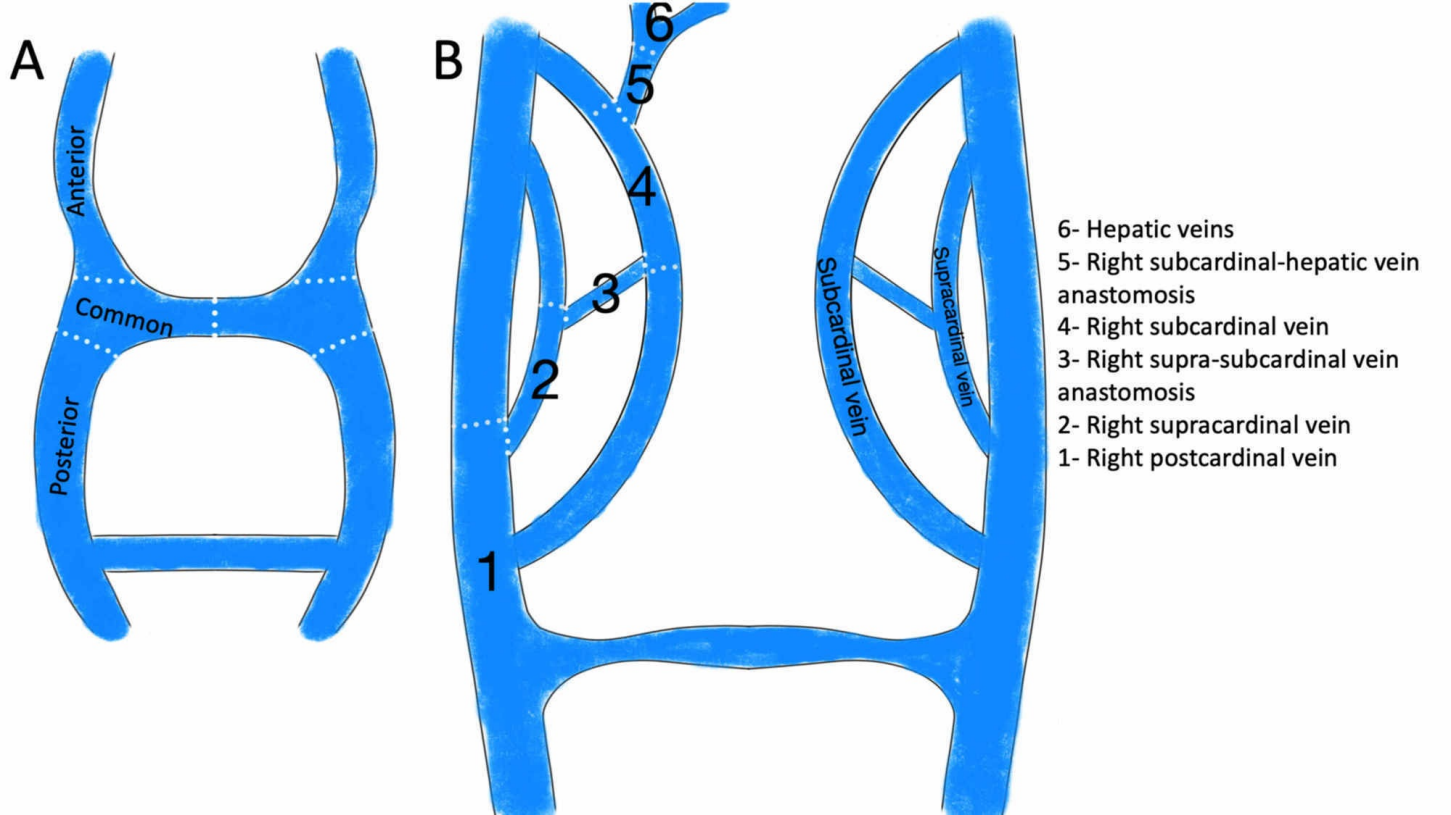
Normal embryological formation of IVC (A) Bilateral posterior cardinal veins drain the lower portion of the embryo. (B) As the left-sided major veins regress, IVC develops from six segments from caudal to cranial. IVC, inferior vena cava

The embryogenesis of IVC starts with bilateral posterior cardinal veins draining the lower portion of the embryo with several anastomoses (Figure 3A). Right-sided veins continue to develop, and left-sided major veins regress. As a consequence, IVC develops from six segments from caudal to cranial: right posterior cardinal vein, right supra-cardinal vein, right supra-sub cardinal vein anastomosis, right sub-cardinal vein, right sub-cardinal vein to hepatic veins anastomosis, and hepatic veins (Fig. 3B). The rest of the major embryological veins shown in Figure 3B regress or form abdominal visceral veins [3]. In our case, the right-sided veins regressed and the left-sided major veins developed into the left-sided IVC with a hemiazygos continuation to left-sided SVC.

Caval anomalies usually do not require treatment. They may be of importance for the planning of vascular intervention or surgery. 

## Conclusions

Congenital anomalies of the caval system are not uncommon and usually do not require treatment. We present a very rare congenital anomaly involving both SVC and IVC, a left-sided IVC with hemiazygos continuation to left SVC in the absence of right SVC. 
